# Vasorelaxant Effect of Moroccan *Cannabis sativa* Threshing Residues on Rat Mesenteric Arterial Bed is Endothelium and Muscarinic Receptors Dependent

**DOI:** 10.1155/2023/1265103

**Published:** 2023-04-20

**Authors:** Youssef Mahou, Alae Chda, Nour Eddine Es-Safi, Angela Tesse, Nezha Fettoukh, Aziz El Bouri, Hamid Stambouli, Kaouakib El Abida, Rachid Bencheikh

**Affiliations:** ^1^LBM2B, FST, USMBA, Fes, Morocco; ^2^Mohammed V University in Rabat, LPCMIO, Materials Science Center (MSC), ENS, Rabat, Morocco; ^3^Nantes Université, INSERM, CNRS, l'Institut du Thorax, Nantes 44007, France; ^4^Institut de Criminalistique de La Gendarmerie Royale, BP 6597 Rabat-Instituts, Rabat, CP 10000, Morocco

## Abstract

**Introduction:**

Ethanolic fraction of Moroccan *Cannabis sativa* threshing residues (EFCS) was evaluated for its vasorelaxant activity. The current work aims to identify the active metabolites in the ethanolic fraction of the EFCS and illustrate their mechanism of action.

**Methods:**

Free radical scavenging capacity of EFCS was assessed using DPPH method. The EFCS vasodilation activities in phenylephrine-precontracted isolated rat mesenteric arterial beds were investigated in presence of L-NAME (nitric oxide synthase inhibitor), indomethacin (cyclooxygenase inhibitor), potassium channel blockers (namely tetraetylamonium, barium chloride, and glibenclamide), and atropine. Nitric oxide vascular release was measured by electron paramagnetic resonance (EPR) using a spin trap in rat aortic rings.

**Results:**

EFCS induced dose-dependent vasorelaxation on mesenteric vascular bed. Incubation of the preparations with L-NAME, ODQ (a soluble guanylyl cyclase inhibitor), or potassium channel blockers reduced the fall of perfusion pressure caused by EFCS. Endothelial denudation or atropine abolished the EFCS's vasorelaxant effect, suggesting involvement of muscarinic receptors and endothelium-relaxing factors. The extract induced nitric oxide release in aortic rings in a similar manner as acetylcholine suggesting an effect of EFCS on the muscarinic receptor and the conductance arteries. Chemical investigation of EFCS identified potential active components namely apigenin and derivatives of luteolin skeleton and also additional components such as neophytadiene, squalene, and *β*-sitosterol. In conclusion, the vasorelaxant effect of EFCS on rat mesenteric arterial bed, which is dependent of muscarinic receptor activation, nitric oxide, and EDHF, can account for potential therapeutic use against high blood pressure related cardiovascular diseases.

## 1. Introduction

Hypertension is a serious cardiovascular complication and is a leading cause of death in adults worldwide [1]. Hence, modulation of arteries' tone is very important to regulate blood flow and pressure in the whole body.

Endothelial cells play a main role in vascular tone control through muscarinic receptor activation [2] and release of several factors such as nitric oxide (NO), prostacyclin (PGI_2_), and endothelium-derived hyperpolarizing factor (EDHF) [3]. These factors illicit relaxation of adjacent smooth muscle cells in the arteries. NO and PGI_2_ may induce the relaxation of arteries by stimulating guanosine 3′, 5′ cyclic monophosphate (cyclic GMP) and adenosine 3′, 5′ cyclic monophosphate (cyclic AMP) productions [3, 4] reducing cytoplasmic Ca^2+^ in vascular smooth muscle cells and decreasing Ca^2+^ sensitivity of the contractile apparatus. These vasodilators may also open K^+^ channels either directly or through their respective second messengers [5]. The endothelium relaxation independent of NO and PGI_2_ is imputed to EDHF which may also act through activation of K^+^ channels opening with subsequent hyperpolarization of smooth muscle cells [3].

Many studies have reported that the vasodilator effect of medicinal plants is attributed to endothelial function or endothelium integrity improvement through muscarinic receptors activation [6–8].

Cannabis is one of the oldest medicinal plants reported to be used in traditional medicine [9]. Cannabis has been shown to have therapeutic potential in diseases associated with inflammation, gastrointestinal disturbance, oxidative stress, neurodegenerative diseases, and metabolic diseases such as diabetes [10, 11].

Cannabis plant produces several hundreds of chemical compounds including cannabinoids, a family of specific substances that exert their biological effects by interacting with endogenous cannabinoid receptors [9, 12, 13].

This plant could be classified into drug and nondrug varieties which have as main difference in the content of the psychoactive cannabinoid namely tetrahydrocannabinol (THC). The drug type, best known in Morocco as kif (khardala), contains THC in concentration between 1 and 20% [14]. The nondrug type, commonly referred as industrial hemp, has no psychoactive activities because it contains less than 0.2% of THC [15].

Apart from cannabidiol (CBD) and cannabigerol (nonpsychoactive), *Cannabis sativa* (*C. sativa*) leaves contain other secondary metabolites such as polyphenols, terpenoids, carbohydrates, and saturated and unsaturated fatty acids [9] that may also have beneficial pharmacological effects. Few identified noncannabinoid phenols in cannabis plant include lignans, dihydrophenanthrene derivatives, stilbenoids, and cannabispirans [16]. Furthermore, cannabis contains many flavonoids belonging to the classes of flavones and flavanols [16].

In the latest decade, nonpsychoactive cannabinoids has been a target for pharmaceutical evaluation owing to their new therapeutic applications.

Several studies demonstrated that CBD can relieve ailments caused by a wide spectrum of diseases such as epilepsy neurodegenerative diseases, neuropsychiatric disorders, and cancer diseases [17, 18]. Recently, many beneficial effects of CBD have been evidenced in heart diseases (myocardial infarction, cardiomyopathy, and myocarditis), stroke, and against ischemia/reperfusion injuries. In these pathological conditions, CBD decreased oxidative stress, apoptosis, and damage to organs and also improved the endothelium function [19–21].

On the other hand, polyphenols (flavonoids) and terpenes have been intensively investigated because of the large spectrum in their biological activities and specifically for their cardiovascular beneficial effects [22, 23].

However, unlike other cannabinoids, the direct vascular effects of nonpsychoactive secondary metabolites from cannabis have not been fully investigated. This study was conducted to evaluate the antioxidant and vasorelaxant effects of nonpsychoactive compounds from Moroccan *C. sativa*. We expected that such approach may be a promising way for converting cannabis or hemp processing by-products into novel bioactive ingredients with cardiovascular benefits. To the best of our knowledge, this is the first study conducted on the Moroccan *C. sativa* leaves and inflorescences threshing residues to test their vasorelaxant effect on rat mesenteric arterial bed (MAB) and NO releasing in rat aorta.

## 2. Materials and Methods

### 2.1. Plant Material

The *C. sativa* plant was harvested in the region of Tafrante in Morocco (37°38′38.67396″N, 5°5′20.92272″W) and transported to the national agency of aromatic and medicinal plants (Taounate, Morocco). Since we are interested to study the nonpsychoactive products of *C. sativa*, only the leaves and inflorescences of the female plant were used, without trichomes and seeds. The trichomes were previously eliminated with a traditional threshing method [24] to extract the cannabis resin fully rich in THC, the psychoactive compound.

### 2.2. Extraction

Leaves and inflorescences of dried threshing residues of *C. sativa* were sequentially extracted using a Soxhlet extractor with 300 mL of hexane, dichloromethane, ethyl acetate, ethanol, and water. Extracts were then filtered through the filter paper with pore size 4–12 *μ*m under vacuum; solvent was evaporated using a rotary vacuum evaporator and stored at 4°C until subsequent testing and analyses.

### 2.3. Quantitative Analysis of Total Phenols (TPC) and Total Flavonoids (TFC)

TPC was determined by the Folin–Ciocalteu colorimetric assay [25], then was expressed as milligrams of gallic acid equivalent per gram dry weight. TFC was determined according to the Harborne method [26] and expressed as milligrams of quercetin equivalent per gram dry weight (mg QE/g dw).

### 2.4. Antioxidant Activity

DPPH free radical scavenging activity of ethanolic fraction of *C. sativa* threshing residues (EFCS) was accessed according to Brand-Williams et al. [27].

The inhibition percentage was expressed by the following equation:

(1) $$\begin{array}{c} \%\;inhibition=\left[\left(\frac{Abs\;control-Abs\;sample}{Abs\;control}\right)*100\right]\text{.} \end{array}$$

At least three independent experiments were carried out and expressed as mean ± SEM values.

### 2.5. Animals

Wistar rats (250–350 g) were cared in compliance with the guide for the care and use of laboratory animals, published by the US National Institutes of Health (National Research Council, 2011: Guide for the care and use of laboratory animals. Washington, D.C.: The National Academies Press; 2011). This experimental protocol has received the local Ethics Committee CEFST (Comité d'éthique de la FST) approval under reference number 11/2021/CEFST, March 09, 2021.

### 2.6. Isolated MAB Experiments

MABs were isolated as described by Cheikh et al. [28]. Rats were anesthetized with sodium pentobarbital (50 mg/kg, i. p.). After opening the abdominal cavity through an abdominal midline incision, the superior mesenteric artery was rapidly cannulated with a heparinized hypodermic needle at its origin from the abdominal aorta and immediately flushed with warm Krebs–Henseleit solution. The whole cannulated arterial bed was carefully separated from the intestines by cutting close to the intestinal wall and placed into a Petri dish. Once the MAB was isolated, the Krebs–Henseleit solution was pumped into the MAB using a peristaltic pump (Pharmacia Biotech, USA) at a continuous constant flow of 2 mL/min. The Krebs–Henseleit solution consisted of 118 mmol/L NaCl, 15 mmol/L NaHCO_3_, 4.7 mmol/L KCl, 1.2 mmol/L MgCl_2_, 2.5 mmol/L CaCl_2_, 1.2 mmol/L KH_2_PO_4_, and 11.0 mmol/L glucose and was maintained at 37°C and aerated with carbogen (5% CO_2_ in O_2_; final pH 7.4). The MAB perfusion pressure was continuously measured using a pressure transducer (Capto SP844) and recorded on a universal oscillograph (50–8622, Harvard Apparatus Limited, UK). The basal perfusion pressure was between 6 and 16 mmHg at the beginning of the study and remained stable throughout the experiment.

### 2.7. Effect of EFCS on the Precontracted MAB

After 30 min equilibration, the MAB was constricted by continuous infusion of phenylephrine (PHE) (10–20 *μ*M) using a syringe pump (Orion M365) to produce a consistent PP of 80–100 mmHg [7]. When the plateau of contraction was reached, the endothelium MAB's integrity was confirmed by 5.5 nmol of acetylcholine (ACh) [7, 28]. Cumulative-dose-response curves were started by bolus (10–100 *μ*l) injections of EFCS (10 to 500 *μ*g), before and after incubation with various pharmacological inhibitors. Assuming that a 100% relaxation represents a return to the baseline, relaxations were reported as a percentage of the drop in perfusion pressure.

#### 2.7.1. Role of Endothelium in the Vascular Relaxant Effect of EFCS

The vascular relaxant effect of EFCS was examined on endothelium-denuded preparations (perfusing MAB with distilled water for 5-6 minutes to remove the endothelium) as described by Adaegbo et al. [29]. The absence of functional endothelium was confirmed by the inability of ACh (5.5 nmol) to induce more than 15% relaxation in precontracted vessels with PHE. To verify the integrity of smooth muscle cells, the MAB was challenged with an exogenous donor of NO, sodium nitroprusside (SNP, 10 nmol) [7, 28].

#### 2.7.2. Role of NO/c-GMP in Vascular Relaxant Effect of EFCS

To investigate the involvement of NO/c-GMP pathway in the observed vasorelaxant effect of EFCS, the endothelium-intact MAB was incubated with a NO-synthase inhibitor, N-nitro-L-arginine methyl ester (L-NAME, 100 *μ*M), or a guanylate cyclase inhibitor (ODQ 1 *μ*M), for 20 min, prior precontracting with a lower concentration of PHE (2.5–5 *μ*M) to get a perfusion pressure plateau identical to that obtained in controls [7, 28].

#### 2.7.3. Role of PGI_2_ in Vascular Relaxant Effects of EFCS

To determine the contribution of PGI_2_ in the EFCS's effect, cyclooxygenase inhibitor (indomethacin, 10 *μ*M) was applied to the MAB with intact endothelium for 20 min prior precontraction with PHE.

#### 2.7.4. Role of K^+^ Channels in Vascular Relaxant Effect of EFCS

The MAB was perfused with Krebs–Henseleit solution containing 100 mM of KCl in order to determine whether the vascular relaxant effect of EFCS is caused by membrane hyperpolarization. Under such condition, the preparation is rather depolarized, preventing any membrane hyperpolarisation and vascular relaxation due to K^+^ channels opening. Therefore, the possibility that EFCS may act as an opener of K^+^ channels was explored by changing the extracellular concentration of K^+^ from 4.7 mM to 100 mM KCl by equimolar replacement of NaCl with KCl in the Krebs–Henseleit solution.

Three potassium channel inhibitors namely tetraethylamonium (TEA) (Ca^2+^-activated K^+^ channel inhibitor), glibenclamide (ATP-sensitive K^+^ channel inhibitor), and barium chloride (BaCl_2_) (inward rectifying potassium channels blocker) were used in a new series of experiments in endothelium-intact MAB to confirm the potential role of K^+^ channels in the EFCS's activity.

#### 2.7.5. Role of Muscarinic Receptors in the Vascular Relaxant Effect of EFCS

To assess if EFCS produced vascular relaxation through the activation of muscarinic receptors, endothelium-intact MAB was incubated with 1 *μ*M atropine (a muscarinic receptor antagonist) for 20 min before the exposure to the EFCS.

#### 2.7.6. Implication of NO in Vascular Relaxant Effect of EFCS

To assess the ability of EFCS to induce NO release in rat vessels, we measured the NO production by electron paramagnetic resonance (EPR) in conductance arteries, the rat aortic rings, exposed or not to ACh (1 *μ*M) and/or EFCS (100 *μ*g/ml), or to the NOS inhibitor L-NAME (0.1 M) as negative control. Aortas were briefly incubated for 45 min at 37°C in a Krebs–Hepes colloid solution with Na-DETC (Sigma-Aldrich) mixed with FeSO4 as a spin trap for NO detection. Each sample was then instantly frozen in liquid nitrogen and analyzed using an EPR Miniscope MS5000 in a Dewar flask at 77°K (Frieberg Instruments, Germany). The instrument settings were microwave power of 10 MW, 1 mT of amplitude modulation, 100 kHz modulation frequency, sweep time of 150 s, and 3 scans. Data were obtained by measuring the overall peak amplitude of the generated spectra and normalizing it to the sample's dry weight in arbitrary units (A.U.) [30].

### 2.8. Phytochemical Analysis

#### 2.8.1. GC-MS Analysis

Analysis of the *C. sativa* ethanolic extract volatile compounds was completed on an Agilent 8890 GC apparatus coupled to an Agilent GC/MSD 5977B mass spectrometry detector. Sample was injected using a 7693A autosampler. A split mode injection was used with a split ratio of 50 : 1. Helium was used as carrier gas, and the injector temperature was set at 250°C. Compounds were separated on a HP-5MS 30 m, 0.25 mm, and 25 *μ*m capillary column. The column oven temperature program started at 60°C for 2 min then ramped from 60 to 295°C during 15 min at a rate of 15°C/min. Results and data were recorded and monitored using Mass Hunter workstation acquisition software.

#### 2.8.2. High-Performance Liquid Chromatography Coupled with Diode-Array Detection and Electrospray Ionization Tandem Mass Spectrometry (HPLC-DAD-MS) Analysis

In order to investigate the nonvolatile phytoconstituents extracted with ethanol from *C. sativa*, an aliquot volume was injected into a reverse phase HPLC apparatus coupled to both UV-visible and mass spectrometry detectors. The LC-DAD-ESI-MS system consisted of a ThermoFinnigan liquid chromatography apparatus, a UV-visible Thermo Surveyor photodiode array detector, and an ion trap LCQ Deca Thermo Finnigan mass spectrometry detector equipped with an electrospray ionization source. Nitrogen was used as nebulizer gas and helium was used as collision gas. Separation was performed on a Merck Hibar® HR Purospher® STAR RP-18 end capped UHPLC column (2.1 × 150 mm). Data were acquired using Xcalibur software. Compounds were detected and tentatively identified from their UV-visible spectra and their MS, MS/MS spectra recorded in the negative ion mode.

### 2.9. Chemicals and Drugs

Distilled water was used to prepare daily ACh, L-NAME, PHE, TEA, glibenclamide, BaCl_2_, and atropine which were kept on ice until their use. Indomethacin was dissolved in NaHCO_3_ (150 mM). ODQ was dissolved in dimethyl sulfoxide (DMSO). Drugs were purchased from Sigma-Aldrich.

### 2.10. Statistical Analysis

All values were expressed as mean ± SEM. For dose-response curves and NO EPR measurements, a one or two way ANOVA analysis for repeated measurements, followed by the Bonferroni's post hoc test were applied. A difference was considered statistically significant at *p* < 0.05. Statistical analyzes were assessed using GraphPad Prism 5.0 software.

## 3. Results

### 3.1. Antioxidant Effect of EFCS

The results obtained from the evaluation of the antioxidant effect by the DPPH method as well as total phenolic content (TPC), and total flavonoid content (TFC) of ethanolic extract is reported in Table 1 below.

The percentage inhibition of antioxidant activity by trapping free radicals was about 43.08 ± 0.65, while the total content of phenols for TPC and TFC were about 8.43 ± 0.28 mg GAE/g dw and 3.13 ± 0.43 mg GAE/g dw, respectively (Table 1), with DPPH activity between 41.44% and 24.22%. In all investigated parameters, the highest values were found in the ethanolic extract and decreased in the following order: ethanol, hexane, dichloromethane, ethyl acetate, and water.

### 3.2. Vasorelaxant Effect and Mechanism of Action of EFCS

**Figure 1:**
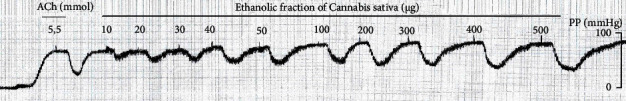
Original graphical recording showing the dose-dependent effect of EFCS extract (10–500 *μ*g) on PP of the MAB precontracted with PHE (10–20 *μ*M).

The bolus injections of EFCS (10–500 *μ*g) caused dose-dependent relaxation of endothelium-intact MAB, which was detectable as a decrease of the perfusion pressure (Figure 1). The maximal relaxation was about 74.17 ± 1.44% of perfusion pressure (PP) decrease and occurred with the perfusion of a solution containing 500 *μ*g of EFCS while the EC_50_ (the dose of EFCS that provides 50% of fall in pressure) was about 47,72 ± 5,664 *μ*g (*n* = 10).

**Figure 2:**
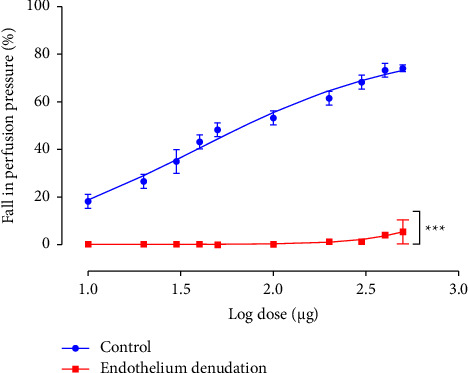
Effects of EFCS on PP of MABs precontracted with PHE (10–20 *μ*M) before and after endothelium denudation. Values are expressed as mean ± SEM (*n* = 10 with and *n* = 6 without endothelium). ^*∗∗∗*^*p* < 0.001 significantly different from the control curve with endothelium.

To investigate the involvement of endothelium in the EFCS's vasorelaxation, new experiments were performed on endothelium-denuded preparations. The removal of endothelium abolished radically the vasorelaxant responses of EFCS (Figure 2), indicating that EFCS-induced vasorelaxation was totally dependent on the endothelial function (*n* = 6).

Under the same experimental conditions, SNP (exogenous NO-donor) confirms the integrity of the smooth muscle cells of the MAB. This verification excludes a possible alteration of smooth muscle cells and highlights that the response to EFCS extract was attenuated because of the destruction of endothelium.

**Figure 3:**
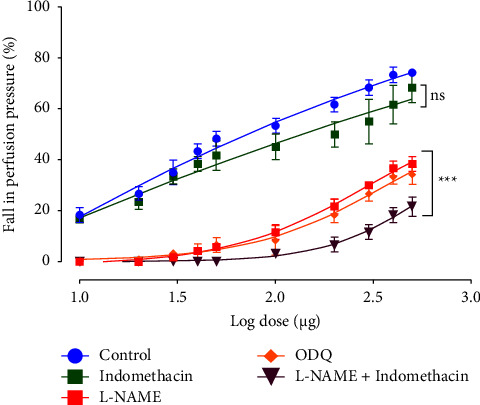
Effects of EFCS extract on rat MAB precontracted with PHE (10–20 *μ*M) in the absence or in the presence of indomethacin (10 *μ*M), L-NAME (100 *μ*M), ODQ (1 *μ*M) or L-NAME + indomethacin. Values are expressed as mean ± SEM (*n* = 10 for control curve without inhibitors and *n* = 6 for all others groups). ns: not significant (control vs indomethacin); ^*∗∗∗*^*p* < 0.001 (control vs L-NAME, ODQ, or L-NAME plus indomethacin).

Interestingly, under NO-sGC blockade, the EFCS-induced vasorelaxation was significantly attenuated (Figure 3) suggesting that response to EFCS was mediated mainly by the NO-sGC-dependent signalling.

To investigate if PGI_2_ mediated the EFCS-induced vasorelaxation we used indomethacin (10 *μ*M) as an inhibitor of cyclooxygenase, an enzyme responsible for synthesis of PGI_2_ in endothelium. As shown in Figure 3, there was no significant reduction in the EFCS-induced vasorelaxation by indomethacin, suggesting that this effect was independent of PGI_2_. However, the simultaneous inhibition of NO and PGI_2_ with both L-NAME and indomethacin seems to reduce the EFCS-induced vasorelaxation further and in a significant manner (Figure 3).

**Figure 4:**
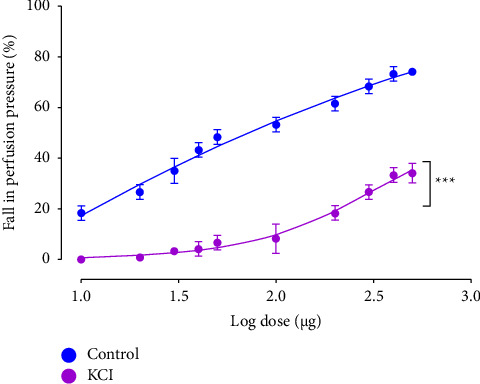
Effects of the EFCS on the fall of PP of the rat MAB precontracted either with PHE (control) or with high potassium medium (experiment). Values are expressed as mean ± SEM (*n* = 10 for control curve and *n* = 6 for high potassium medium group). ^*∗∗∗*^*p* < 0.001 significantly different from the respective control.

To find out the possible implication of K^+^ channels opening in the EFCS's vasorelaxation, experiments were performed on preparations precontracted with Krebs high K^+^ (100 mM KCl). As shown in Figure 4, vasorelaxation caused by EFCS on MAB vessels was significantly diminished on preparations depolarized and precontracted with high K^+^ comparing to the preparations precontracted with PHE, suggesting the involvement of activated K^+^ channels associated to EDHF relaxation.

**Figure 5:**
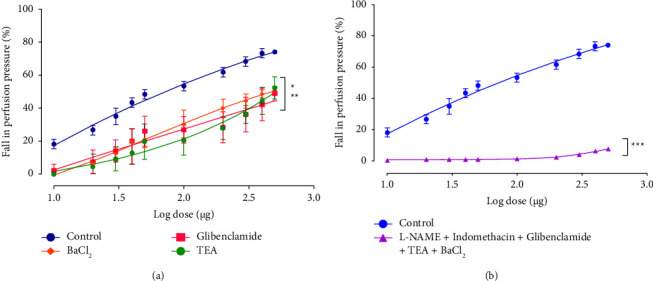
Effects of EFCS extract on PP of rat MAB precontracted with PHE in the absence and presence of of K^+^ channel blockers (10 mM of TEA, 1 *μ*M of glibenclamide, and 100 *μ*M of BaCl_2_) (a) and L-NAME + indomethacin + K^+^ channels blockers (b) Values are expressed as mean ± SEM (*n* = 10 for control curve without inhibitors and *n* = 6 for all others groups). ^*∗*^*p* < 0.05 (control vs. BaCl_2_); ^*∗∗*^*p* < 0.01 (control vs. glibenclamide or TEA); ^*∗∗∗*^*p* < 0.001 (control vs. L-NAME + Indomethacin + glibenclamide + TEA + BaCl_2_).

Furthermore, the involvement of these channels was assessed in the presence of three K^+^ channel inhibitors namely TEA (an inhibitor of Ca^2+^-activated K^+^ channels), glibenclamide (an inhibitor of ATP-sensitive K^+^ channels), and BaCl_2_ (a blocker of inwardly rectifying potassium channels). The obtained data revealed that preincubation of MAB with 10 mM of either TEA, glibenclamide, or BaCl_2_ caused an inhibitory effect on EFCS-induced vasorelaxation suggesting that the observed EFCS vasorelaxant effect requires at least the involvement of the three types of K^+^ channels (Figure 5(a)). The inhibition of these channels simultaneously in the presence of L-NAME and indomethacin completely abolished the EFCS-induced relaxation (Figure 5(b)).

**Figure 6:**
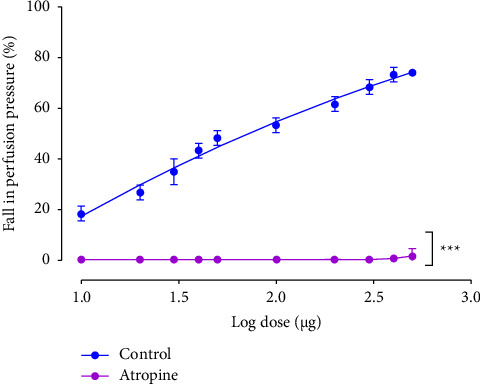
Graph showing the inhibition of the EFCS effect on rat MABs precontracted with PHE (10–20 *μ*M) in presence of atropine (1 *μ*M). ^*∗∗∗*^*p* < 0.001 significantly different from the respective control. Values are expressed as mean ± SEM (*n* = 10 for control curve; *n* = 6 for curves in presence of atropine).

To verify if EFCS act through muscarinic receptors, the MAB was incubated with atropine.  Figure 6 shows that 1 *μ*M atropine almost completely abolished the relaxant effect of EFCS, suggesting that muscarinic receptors are activated to induce relaxation of MAB when incubated with EFCS. The functionality of the preparation was confirmed by evaluating the vasorelaxation induced by 10 nmol of the NO-donor SNP (data not shown).

**Figure 7:**
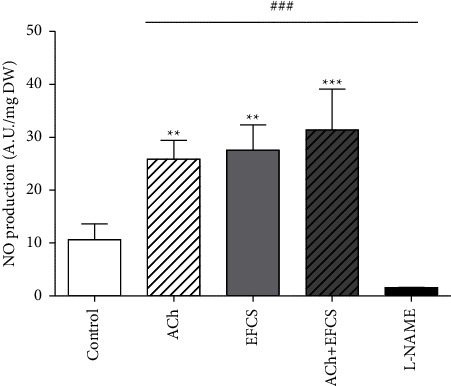
Evaluation of NO production by electronic paramagnetic resonance (EPR) using Fe (DETC)_2_ as spin trap on aorta with or without inhibitor (ACh 1 *μ*M, EFCS 100 *μ*g, alone or in association or L-NAME, 100 *μ*M as negative control). Data are expressed as mean ± SEM (*n* = 4 for each group of values), ^*∗∗*^*p* < 0.01; ^*∗∗∗*^*p* < 0.001 versus control; ^###^*p* < 0.001 versus L-NAME.

To verify if the EFCS is able to induce potentially vascular relaxation in conductance arteries such as aorta, we measured the release of NO in aortic rings by EPR using Fe (DETC)_2_ as spin trap. Indeed, NO in conductance arteries is the main vasorelaxant factor involved. As expected, NO release is significantly increased when the aortic ring is incubated with ACh (1 *μ*M). Interestingly, the vessel is able to release a similar amount of NO if incubated with a solution of spin trap containing 100 *μ*g of EFCS, suggesting eNOS involvement in this effect (Figure 7). If added to ACh, EFCS is not able to improve ulterior NO release induced by ACh (1 *μ*M) suggesting a maximal effect of the EFCS at the dose used. Finally, the use of L-NAME also abolished the basal level of NO released by control vessels suggesting that the signal evaluated is NO of NOS origin in all the groups of aortic rings (Figure 7).

### 3.3. Phytochemical Analysis of EFCS

The phytochemical composition of the studied *C. sativa* ethanolic extract was explored through hyphenated coupled chromatographic techniques. While the nonvolatile fraction of the extract was explored through high performance liquid chromatography coupled to both mass spectrometry and diode array detectors, the volatile compounds were identified by gas chromatography coupled to mass spectrometry (GC-MS). The generated data from both analyses are discussed below.

The generated data for the volatile metabolites representing the major detected compounds by GC-MS are gathered in the Table 2 below. The identification of the components was made by comparing the mass spectra of each constituent with those stored in the NIST MS Search 2.4. The confirmation was done by comparing their Kovats retention index with those reported in the literature. The calculation of the Kovats index was made based on a linear interpolation of the retention time of homologous series of n-alkanes (C8–C20; C21–C40) under the same GC-MS operating conditions and column used to obtain the peaks.

**Figure 8:**
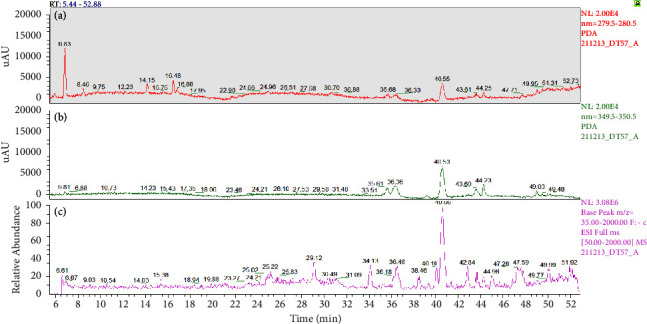
HPLC chromatographic profiles of ethanolic *C sativa* fraction recorded at 280 nm (a), 350 nm (b), and negative ESI-MS detection (c).

The nonvolatile phytoconstituents identified by LC-MS/MS in the *Cannabis* extracts are listed in Table 3 along with their retention time, *m*/*z* values, and MS/MS ion fragment. The obtained chromatographic profiles are shown in Figure 8.

Five compounds were tentatively identified as apigenin and derivatives of luteolin skeleton. Thus, the presence of neophytadiene, squalene, *β*-sitosterol, apigenin, and luteolin derivatives in high amounts in EFCS could be responsible for the antioxidant and the vasorelaxant effects of EFCS.

## 4. Discussion

The goal of this study focused on understanding the actions of nonpsychoactive *Cannabis* compounds on vascular tone modulation. To our knowledge, this is the first study that demonstrates how EFCS of *C. sativa* threshing residues causes vasorelaxation in rat MAB, which is dependent on muscarinic receptor activation, endothelial activity, NO, and potassium channel activity modulation.

The *C. sativa* threshing ethanolic extract contained a high level of total polyphenols (TP) and total flavonoids (TF), 8.43 ± 0.28 mg GAE/g dw and 3.13 ± 0.43 mg GAE/g dw, respectively. In agreement with a previous study reporting similar levels of TP and TF content of aerial parts of mature hemp that ranged from 5.85 to 9.25 mg GAE/g dw and from 1.83 to 5.21 mg CE/g dw, respectively [31].

Our data agreed with a study by Kitrytė et al. [32] demonstrating the highest antioxidant capacity in the ethanolic fraction harvested from *C. sativa* threshing residues. Numerous epidemiological and in vitro investigations suggested that polyphenol-rich herbal medicine extracts play a significant role in maintaining vascular health and preventing cardiovascular diseases [22, 23]. Indeed previous studies have shown that nonpsychoactive compounds from *Cannabis* have antioxidant effects [16, 33]. When confronted by cardiovascular diseases, antioxidant therapy has been recognized as a reasonable strategy to improve vascular function [34, 35]. The scavenging effect displayed by *Cannabis* threshing compounds could be due to the high content of total polyphenols and flavonoids in EFCS.

Furthermore, our study demonstrates that EFCS induced a dose-dependent vasorelaxation on preconstricted MAB with a pEC50 in the midmicromolar range. Similar findings have been reported in the rat mesenteric artery with other medicinal plant extracts [7, 36] and also with *Cannabis-*derived products [20, 21].

The endothelium is well known to play a main role in the vasorelaxation process and implicates activation of the muscarinic receptor [2, 3]. EFCS-induced vasorelaxation seems to be totally endothelium dependent, since the removal of endothelial cells by water perfusion radically abolished the fall in perfusion pressure. Endothelium-dependent vasorelaxation in mesenteric arteries implicates release of endothelium-derived relaxing factors, such as NO, PGI_2_, and EDHF [3]. The NO is synthesized from L-arginine by endothelial-NOS. L-NAME, a L-arginine analog is able to inhibit the 3 isoforms of NOS in a nonspecific way. PGI_2_ is synthesized by endothelial cyclooxygenase 1 (COX1) using arachidonic acid as substrate [3], and we can inhibit the PGI_2_ production using indomethacin, a nonselective COX inhibitor able to inhibit also the inducible isoform (COX2).

We next investigated which endothelium-derived relaxing factors, namely NO, PGI_2_, and EDHF was/were implicated in EFCS-induced vasorelaxation. Here again, the incubation of the MAB with L-NAME attenuated significantly but not totally the EFCS-induced vasorelaxation. This finding suggests that the latter is at least NO dependent and that the remaining part is caused by additional endothelium relaxing substances like PGI_2_ and/or EDHF. Also, we observed that NO inhibition release by L-NAME potentiated the PHE-induced contraction, explaining the reason why we had lowered PHE concentrations from 10-20 *μ*M to 2.5–5 *μ*M to achieve a PP close to 80–100 mmHg.

This is consistent with numerous other studies demonstrating that NO inhibition enhances the MAB's adrenoceptor response. [29, 37]. Moreover, the blockage with ODQ of the NO second messenger, the cGMP, affected significantly EFCS's effect, confirming that this vasorelaxation requires at least the NO-cGMP pathway.

We tried to assess if PGI_2_ mediated the EFCS-induced vasorelaxation by incubating the intact MAB with 10 *μ*M indomethacin to inhibit COX. In this condition, the induced vasorelaxation was not significantly modified, suggesting a marginal contribution of PGI_2_ in the relaxation. In contrast, the association of indomethacin and L-NAME showed a significant attenuation of the vasorelaxation, suggesting that COX inhibition potentiates the NO inhibition release. Similar findings were reported in our previous studies [7, 28].

In order to investigate the involvement of EDHF in EFCS's effect, vasorelaxation mediated by the opening of K^+^ channels was prevented by precontracting MABs with a solution containing high concentration of KCl (100 mM). In this condition, the preparation is rather depolarized preventing the K^+^-dependent relaxation [38]. The EFCS-induced vasorelaxation on MABs precontracted with high potassium preparations was significantly diminished compared to the preparations precontracted with PHE. This suggests that activation of K^+^ channels is also involved in the *C. sativa* extract effect.

To confirm the involvement of K^+^ channels in the EFCS's vasorelaxant effect, additional assays were performed in the presence of 3 potassium channel inhibitors (e.g., TEA, glibenclamide, and BaCl_2_). Our results revealed that simultaneous preincubation of the MAB with these blockers, drastically reduced the EFCS-induced vasorelaxation, suggesting the involvement at least of these 3 K^+^ channels in the vascular effect. When the MAB was incubated simultaneously with L-NAME, Indomethacin, and the latter blockers of the three types of K^+^ channels, the EFCS's effect was completely abolished, confirming that the observed relaxation is totally endothelium dependent and mediated at least by NO production and K^+^ channels opening corresponding to EDHF.

Previous studies already reported that nonpsychoactive cannabinoid induces vasorelaxation in different vascular beds, through cannabinoid receptors and endothelium activation [20, 21]. In this study, we found that EFCS induces arterial dilation via NO and EDHF, but the first target of our extract seems to be muscarinic receptor since atropine abolished this vasorelaxation. This result is in concordance with our previous study [7] and others [22, 23] who showed that arterial relaxation by medicinal plant extracts can be mediated by the activation of muscarinic receptors.

In this study, we also confirmed that the increased level of NO release by aortic rings when incubated with EFCS is similar to that of ACh incubation, suggesting the implication of eNOS activation and muscarinic receptors in the effect also in conductance arteries. This finding suggests that the extract is potentially able to improve endothelial function in both resistance and conductance arteries.

The phytochemical analysis initiated on our extract showed the presence of compounds pertaining to different families such as alkanes/hydrocarbons, fatty acids derivatives, sterols, and noncannabinoids phenolic compounds among others, which were previously reported in *C. sativa* extracts [9, 12, 13, 16]. Differences and discrepancies which may be evidenced between the phytochemical profile observed in this work and those previously reported may be due to different conditions such as climate, soil composition, date and place of harvest, and orientation.

The GC-MS analysis of EFCS is also notably significant because it confirms that our extract does not almost contain the more known cannabinoids as CBD or CBG, even not THC, but shows that it contains high levels of undecane, *neophytadiene*, squalene, *β*-sitosterol, heptacosane, and 17-pentatriacontene. *β*-sitosterol and squalene have been previously reported in *Cannabis* species [39, 40].

The HPLC analysis confirmed the presence of phenolic compounds in EFCS. Among the nine compounds detected, five have been tentatively identified from their UV absorbance and mass spectral characteristics. The five compounds were concluded to pertain to the flavonoids' family [41–44] as it was clearly observed on the chromatographic profile recorded at 350 nm where the area of these five peaks washigher than those recorded at 280 nm. The five compounds were tentatively identified as apigenin and derivatives of luteolin skeleton.

In addition to these compounds, we tried to check for other metabolites such as condensed tannins. Their occurrence was assayed through phloroglucinolysis reaction [41] which demonstrated the absence of such derivatives in the investigated extract.

Phytosterols such as, *β*-sitosterol, have been reported to be an efficient antioxidant and free radical scavenger [42] as well as squalene [43] and neophytadiene [44].

Apigenin and luteolin have been already detected and isolated from *Cannabis* [45]. In addition to their well-documented antioxidant effect, those flavonoids have been described to exhibit endothelium-dependent vasorelaxant effect [22, 23]. Also, our results are in concordance with those of Abdallah et al. [6] who show that *β*-sitosterol induces significant vasodilation activities that were blocked by either endothelial denudation or L-NAME (NOS inhibitor), pointing towards a role of endothelial NO in their activities.

Thus, the presence of neophytadiene, squalene, *β*-sitosterol, apigenin, and luteolin derivatives in high amounts in EFCS could be responsible for the antioxidant and the vasorelaxant effects of EFCS.

The summary of our study on precontracted isolated rat MAB shows that the effect of EFCS was drastically diminished by NO-synthase inhibition and potassium channels blockers, and totally abolished by endothelium removal. These results are relevant for the better understanding of *C. sativa* threshing compounds beneficial actions on the cardiovascular system, and could suggest the use of the extract to improve endothelial functions. Since hypertension can be due to increased peripheral resistance and endothelial dysfunction, and since vasodilator and antioxidant activities of EFCS were proven only *ex vivo*, a follow up *in vivo* studies on hypertensive experimental animal models are required in order to assess the *in vivo* effect of EFCS's compounds and to prove their potential therapeutic use against high-blood-pressure-related cardiovascular diseases.

## Figures and Tables

**Table 1:** Results of quantitative analysis of phenols content (TPC), flavonoids content (TFC), and DPPH activity of the soxhlet extracts from threshing *C. sativa.*

Soxhlet fraction	TPC (mg GAE/g DW)	TFC (mg QE/g DW)	DPPH activity (%)
Ethanol	8.43 ± 0.28	3.13 ± 0.43	43.08 ± 0.65

**Table 2:** Major volatile compounds detected in *C. sativa* ethanolic extract determined through GC-MS analysis.

Peak number	Retention time	Compound^1^	Kri-exp^2^	Kri-theo^3^	R.A (%)
1	6.86	Undecane	1090	1100	0.57
2	12.45	Nd	—	—	0.75
3	13.25	Neophytadiene	1843	1838	1.38
4	13.84	Palmitic acid methyl ester	1927	1924	5.96
5	14.53	Digitoxin	1967	N.A^4^	1.20
6	14.99	Nd	—	—	14.47
7	15.13	Methyl stearate	2130	2128	4.43
8	16.13	Nd	—	—	7.58
9	16.69	Nd	—	—	5.96
10	16.89	17-pentatriacontene	2531	N.A^4^	2.55
11	17.22	Nd	—	—	5.82
12	17.56	Nd	—	—	9.65
13	17.75	Heptacosane	2696	2700	6.11
14	18.29	Nd	—	—	4.67
15	18.65	Nd	—	—	5.80
16	18.89	Nd	—	—	4.04
17	19.16	Squalene	2842	2847	9.91
18	21.39	Nd	—	—	2.16
19	21.57	Nd	—	—	3.08
20	22.57	Nd	—	—	2.12
21	24.65	*β*-sitosterol	3200	3197	1.78

^1^Compounds: these are listed in order of elution on an HP-5MS column; N.A: not available; ^2^Kri-exp: Kovats retention index on HP-5MS obtained experimentally; ^3^Kri-theo: Kovats retention index from the literature; ^4^Identification by comparison with mass fragmentation patterns from the literature; R.A: relative amounts (%) were obtained by peak areas normalization.

**Table 3:** Major nonvolatile components identified by HPLC coupled to both DAD and MS detectors in the *C. sativa* ethanolic extract.

Peak	Rt (min)	*λ*_max_ (nm)	(M–H)^−^	Major MS/MS product ions	Identification
1	6.83	300	1153	1001 (60), 983 (100)	Nd
2	8.46	295	Nd	Nd	Nd
3	14.15	280	nd	Nd	Nd
4	16.48	266	443	375 (95), 335 (100), 219 (96)	Nd
5	35.61	350	447	357 (58), 327 (100)	Luteolin *C*-glucoside
6	36.36	350	593	473 (100), 357 (58), 327 (54)	Luteolin C-rhamnoglucoside
7	40.53	336	577	413 (100)	Apigenin *C*-rhamnoglucoside
8	43.60	350	607	443 (100), 341 (42)	Methoxy luteolin *C*-rhamnoglucoside
9	44.23	347	461	341 (100)	Methoxy luteolin *C*-glucoside

## Data Availability

Data in supplementary information file concern the repeated individual measurements of each parameter investigated in this study. Data will be made available upon request.

## Supplementary Materials

Supplementary Materials (1) The influence of EFCS in the absence or in the presence of L-NAME. (2) The influence of EFCS in the absence or in the presence of ODQ. (3) The influence of EFCS in the absence or in the presence of Indomethacin. (4) The influence of EFCS in the absence or in the presence of Indomethacin + L-NAME. (5) The influence of EFCS in the absence or in the presence of K^+^. (6) The influence of EFCS in the absence or in the presence of TEA. (7) The influence of EFCS in the absence or in the presence of BaCl. (8) The influence of EFCS in the absence or in the presence of Glibenclamide. (9) The influence of EFCS in the absence or in the presence of L-NAME + Indo + TEA + Gly + BaCl.
