# Supplementary material for: Vasorelaxant Effect of Moroccan Cannabis sativa Threshing Residues on Rat Mesenteric Arterial Bed is Endothelium and Muscarinic Receptors Dependent
Source: Evid Based Complement Alternat Med. 2023 Apr 20;2023:1265103. doi: 10.1155/2023/1265103 (PMC10139821; doi:10.1155/2023/1265103)
Supplement: Supplementary Materials — (1) The influence of EFCS in the absence or in the presence of L-NAME. (2) The influence of EFCS in the absence or in the presence of ODQ. (3) The influence of EFCS in the absence or in the presence of Indomethacin. (4) The influence of EFCS in the absence or in the presence of Indomethacin + L-NAME. (5) The influence of EFCS in the absence or in the presence of K+. (6) The influence of EFCS in the absence or in the presence of TEA. (7) The influence of EFCS in the absence or in the presence of BaCl. (8) The influence of EFCS in the absence or in the presence of Glibenclamide. (9) The influence of EFCS in the absence or in the presence of L-NAME + Indo + TEA + Gly + BaCl. [file 1265103.f1.docx]

| the influence of EFCS in the absence or in the presence of L-NAME | | | | | | | | | | | | | | | | | | | | | | | | | | | | | | | | | | | | | | | | | |  | | |
| --- | --- | --- | --- | --- | --- | --- | --- | --- | --- | --- | --- | --- | --- | --- | --- | --- | --- | --- | --- | --- | --- | --- | --- | --- | --- | --- | --- | --- | --- | --- | --- | --- | --- | --- | --- | --- | --- | --- | --- | --- | --- | --- | --- | --- |
|  | | | | | | |  | | | | | | | |  | | | | | | | | | |  | | | | | | | | |  | | | | |  | | |  | | |
| Dose (μg) | | | | | | | L-NAME | | | | | | | | | | | | | | | | | | | | | | | | | | | | | | | | | | | | | |
| 10 | | | | | | | 0 | | | | | | | | 0 | | | | | | | | | | 0 | | | | | | | | | 0 | | | | | 0 | | | 0 | | |
| 20 | | | | | | | 0 | | | | | | | | 0 | | | | | | | | | | 0 | | | | | | | | | 0 | | | | | 0 | | | 0 | | |
| 30 | | | | | | | 2,5 | | | | | | | | 0 | | | | | | | | | | 2,5 | | | | | | | | | 2,5 | | | | | 2,5 | | | 0 | | |
| 40 | | | | | | | 5 | | | | | | | | 2,5 | | | | | | | | | | 5 | | | | | | | | | 5 | | | | | 5 | | | 2,5 | | |
| 50 | | | | | | | 5 | | | | | | | | 5 | | | | | | | | | | 7,5 | | | | | | | | | 5 | | | | | 5 | | | 7,5 | | |
| 100 | | | | | | | 8 | | | | | | | | 17 | | | | | | | | | | 10 | | | | | | | | | 12 | | | | | 13 | | | 10 | | |
| 200 | | | | | | | 20 | | | | | | | | 20 | | | | | | | | | | 25 | | | | | | | | | 20 | | | | | 25 | | | 20 | | |
| 300 | | | | | | | 30 | | | | | | | | 30 | | | | | | | | | | 30 | | | | | | | | | 30 | | | | | 30 | | | 30 | | |
| 400 | | | | | | | 35 | | | | | | | | 34,5 | | | | | | | | | | 35 | | | | | | | | | 35 | | | | | 40 | | | 40,5 | | |
| 500 | | | | | | | 35 | | | | | | | | 34 | | | | | | | | | | 40 | | | | | | | | | 40 | | | | | 41 | | | 40 | | |
|  | | | | | | |  | | | | | | | |  | | | | | | | | | |  | | | | | | | | |  | | | | |  | | |  | | |
| the influence of EFCS in the absence or in the presence of ODQ | | | | | | | | | | | | | | | | | | | | | | | | | | | | | | | | | | | | |  | | | | |  | | |
|  | | | |  | | | | | | | | |  | | | | | | | | |  | | | | | | | | | |  | | | | |  | | | | |  | | |
| Dose (μg) | | | | ODQ | | | | | | | | | | | | | | | | | | | | | | | | | | | | | | | | | | | | | | | | |
| 10 | | | | 0 | | | | | | | | | 0 | | | | | | | | | 0 | | | | | | | | | | 0 | | | | | 0 | | | | | 0 | | |
| 20 | | | | 0 | | | | | | | | | 2,5 | | | | | | | | | 0 | | | | | | | | | | 2,5 | | | | | 0 | | | | | 0 | | |
| 30 | | | | 1,5 | | | | | | | | | 5 | | | | | | | | | 2,5 | | | | | | | | | | 3,5 | | | | | 5 | | | | | 2,5 | | |
| 40 | | | | 1,5 | | | | | | | | | 9,5 | | | | | | | | | 3 | | | | | | | | | | 2,5 | | | | | 5,5 | | | | | 3 | | |
| 50 | | | | 5 | | | | | | | | | 11,5 | | | | | | | | | 5 | | | | | | | | | | 7 | | | | | 8 | | | | | 3,5 | | |
| 100 | | | | 5 | | | | | | | | | 19 | | | | | | | | | 5 | | | | | | | | | | 8 | | | | | 10 | | | | | 3 | | |
| 200 | | | | 15 | | | | | | | | | 21 | | | | | | | | | 20 | | | | | | | | | | 14 | | | | | 20 | | | | | 20 | | |
| 300 | | | | 25 | | | | | | | | | 25 | | | | | | | | | 30 | | | | | | | | | | 24 | | | | | 25 | | | | | 31 | | |
| 400 | | | | 29 | | | | | | | | | 35 | | | | | | | | | 35 | | | | | | | | | | 35 | | | | | 30 | | | | | 36 | | |
| 500 | | | | 29,5 | | | | | | | | | 35 | | | | | | | | | 37,5 | | | | | | | | | | 35 | | | | | 30 | | | | | 38 | | |
|  | | | |  | | | | | | | | |  | | | | | | | | |  | | | | | | | | | |  | | | | |  | | | | |  | | |
| the influence of EFCS in the absence or in the presence of Indomethacin | | | | | | | | | | | | | | | | | | | | | | | | | | | | | | | | | | | | | | | | | |  | | |
|  | | | | | |  | | | | | | | | | |  | | | | | | |  | | | | | | | | | |  | | | | | | |  | |  | | |
| Dose (μg) | | | | | | Indomethacin | | | | | | | | | | | | | | | | | | | | | | | | | | | | | | | | | | | | | | |
| 10 | | | | | | 17,5 | | | | | | | | | | 15 | | | | | | | 17,5 | | | | | | | | | | 17,5 | | | | | | | 15 | | 17,5 | | |
| 20 | | | | | | 20 | | | | | | | | | | 27 | | | | | | | 25 | | | | | | | | | | 20 | | | | | | | 25 | | 22 | | |
| 30 | | | | | | 35 | | | | | | | | | | 30 | | | | | | | 35 | | | | | | | | | | 35 | | | | | | | 30 | | 35 | | |
| 40 | | | | | | 40 | | | | | | | | | | 35 | | | | | | | 40 | | | | | | | | | | 40 | | | | | | | 35 | | 40 | | |
| 50 | | | | | | 45 | | | | | | | | | | 33 | | | | | | | 45 | | | | | | | | | | 45 | | | | | | | 35 | | 47 | | |
| 100 | | | | | | 45 | | | | | | | | | | 40 | | | | | | | 47 | | | | | | | | | | 45 | | | | | | | 40 | | 53 | | |
| 200 | | | | | | 50 | | | | | | | | | | 44 | | | | | | | 55 | | | | | | | | | | 50 | | | | | | | 45 | | 56 | | |
| 300 | | | | | | 50 | | | | | | | | | | 50 | | | | | | | 67 | | | | | | | | | | 50 | | | | | | | 48 | | 65 | | |
| 400 | | | | | | 53 | | | | | | | | | | 60 | | | | | | | 70 | | | | | | | | | | 55 | | | | | | | 60 | | 72 | | |
| 500 | | | | | | 65 | | | | | | | | | | 65 | | | | | | | 75 | | | | | | | | | | 63 | | | | | | | 65 | | 77 | | |
|  | | | | | |  | | | | | | | | | |  | | | | | | |  | | | | | | | | | |  | | | | | | |  | |  | | |
| the influence of EFCS in the absence or in the presence of Indomethacin + L-NAME | | | | | | | | | | | | | | | | | | | | | | | | | | | | | | | | | | | | | | | | | | | | |
|  | | | | | | | |  | | | | | | | | | |  | | | | | | | |  | | | | | | | | |  | | | | | |  | | |  |
| Dose (μg) | | | | | | | | Indomethacin + L-NAME | | | | | | | | | | | | | | | | | | | | | | | | | | | | | | | | | | | | |
| 10 | | | | | | | | 0,1 | | | | | | | | | | 0,1 | | | | | | | | 0,1 | | | | | | | | | 0,1 | | | | | | 0,1 | | | 0,1 |
| 20 | | | | | | | | 0,1 | | | | | | | | | | 0,1 | | | | | | | | 0,1 | | | | | | | | | 0,1 | | | | | | 0,1 | | | 0,1 |
| 30 | | | | | | | | 0,1 | | | | | | | | | | 0,1 | | | | | | | | 0,1 | | | | | | | | | 0,1 | | | | | | 0,1 | | | 0,1 |
| 40 | | | | | | | | 0,1 | | | | | | | | | | 0,1 | | | | | | | | 0,1 | | | | | | | | | 0,1 | | | | | | 0,1 | | | 0,1 |
| 50 | | | | | | | | 0,1 | | | | | | | | | | 0,1 | | | | | | | | 0,1 | | | | | | | | | 0,1 | | | | | | 0,1 | | | 0,1 |
| 100 | | | | | | | | 2,5 | | | | | | | | | | 5 | | | | | | | | 2,5 | | | | | | | | | 3,5 | | | | | | 5 | | | 1,5 |
| 200 | | | | | | | | 5 | | | | | | | | | | 11 | | | | | | | | 4 | | | | | | | | | 5 | | | | | | 10 | | | 5 |
| 300 | | | | | | | | 10 | | | | | | | | | | 15 | | | | | | | | 9 | | | | | | | | | 10 | | | | | | 16 | | | 10 |
| 400 | | | | | | | | 15 | | | | | | | | | | 20 | | | | | | | | 20 | | | | | | | | | 14 | | | | | | 20 | | | 21 |
| 500 | | | | | | | | 17,5 | | | | | | | | | | 25 | | | | | | | | 22 | | | | | | | | | 16,5 | | | | | | 25 | | | 23 |
|  | | | | | | | |  | | | | | | | | | |  | | | | | | | |  | | | | | | | | |  | | | | | |  | | |  |
| the influence of EFCS in the absence or in the presence of K+ | | | | | | | | | | | | | | | | | | | | | | | | | | | | | | | | | | | | |  | | | | |  | | |
|  | | | | |  | | | | | | | | |  | | | | |  | | | | | | | | | | | |  | | | | | |  | | | | |  | | |
| Dose (μg) | | | | | K+ | | | | | | | | | | | | | | | | | | | | | | | | | | | | | | | | | | | | | | | |
| 10 | | | | | 0 | | | | | | | | | 0 | | | | | 0 | | | | | | | | | | | | 0 | | | | | | 0 | | | | | 0 | | |
| 20 | | | | | 0 | | | | | | | | | 2,5 | | | | | 0 | | | | | | | | | | | | 0 | | | | | | 2,5 | | | | | 0 | | |
| 30 | | | | | 2,5 | | | | | | | | | 5 | | | | | 2,5 | | | | | | | | | | | | 3 | | | | | | 5,5 | | | | | 2,5 | | |
| 40 | | | | | 2,5 | | | | | | | | | 7,5 | | | | | 2,5 | | | | | | | | | | | | 3,5 | | | | | | 8,5 | | | | | 2,5 | | |
| 50 | | | | | 5 | | | | | | | | | 10 | | | | | 5 | | | | | | | | | | | | 4 | | | | | | 11 | | | | | 5 | | |
| 100 | | | | | 5 | | | | | | | | | 15 | | | | | 5 | | | | | | | | | | | | 4 | | | | | | 16 | | | | | 5 | | |
| 200 | | | | | 15 | | | | | | | | | 20 | | | | | 20 | | | | | | | | | | | | 14 | | | | | | 20 | | | | | 21 | | |
| 300 | | | | | 25 | | | | | | | | | 25 | | | | | 30 | | | | | | | | | | | | 24 | | | | | | 25 | | | | | 31 | | |
| 400 | | | | | 30 | | | | | | | | | 35 | | | | | 35 | | | | | | | | | | | | 29 | | | | | | 35 | | | | | 36 | | |
| 500 | | | | | 30 | | | | | | | | | 35 | | | | | 37,5 | | | | | | | | | | | | 29 | | | | | | 35 | | | | | 38,5 | | |
|  | | | | |  | | | | | | | | |  | | | | |  | | | | | | | | | | | |  | | | | | |  | | | | |  | | |
| the influence of EFCS in the absence or in the presence of TEA | | | | | | | | | | | | | | | | | | | | | | | | | | | | | | | | | | | | |  | | | | |  | | |
|  | |  | | | | | | | | |  | | | | | | | | | |  | | | | | | | |  | | | | | | | |  | | | | |  | | |
| Dose (μg) | | TEA | | | | | | | | | | | | | | | | | | | | | | | | | | | | | | | | | | | | | | | | | | |
| 10 | | 0 | | | | | | | | | 0 | | | | | | | | | | 0 | | | | | | | | 0 | | | | | | | | 0 | | | | | 0 | | |
| 20 | | 0 | | | | | | | | | 18,33 | | | | | | | | | | 0 | | | | | | | | 0 | | | | | | | | 8,33 | | | | | 0 | | |
| 30 | | 8,33 | | | | | | | | | 18,66 | | | | | | | | | | 1,5 | | | | | | | | 9,33 | | | | | | | | 14,66 | | | | | 2,5 | | |
| 40 | | 16,66 | | | | | | | | | 18,66 | | | | | | | | | | 3 | | | | | | | | 16,66 | | | | | | | | 16,66 | | | | | 5 | | |
| 50 | | 25 | | | | | | | | | 28,66 | | | | | | | | | | 7,5 | | | | | | | | 25 | | | | | | | | 26,66 | | | | | 5,5 | | |
| 100 | | 25 | | | | | | | | | 28,66 | | | | | | | | | | 10 | | | | | | | | 25 | | | | | | | | 26,66 | | | | | 8 | | |
| 200 | | 33,33 | | | | | | | | | 30,16 | | | | | | | | | | 18 | | | | | | | | 35,33 | | | | | | | | 30,16 | | | | | 20 | | |
| 300 | | 40,33 | | | | | | | | | 38,48 | | | | | | | | | | 30 | | | | | | | | 40,33 | | | | | | | | 38,48 | | | | | 30 | | |
| 400 | | 50 | | | | | | | | | 48,33 | | | | | | | | | | 35 | | | | | | | | 53 | | | | | | | | 48,33 | | | | | 32 | | |
| 500 | | 58,33 | | | | | | | | | 53,33 | | | | | | | | | | 45 | | | | | | | | 60 | | | | | | | | 53,33 | | | | | 42,77 | | |
|  | |  | | | | | | | | |  | | | | | | | | | |  | | | | | | | |  | | | | | | | |  | | | | |  | | |
| the influence of EFCS in the absence or in the presence of BaCl | | | | | | | | | | | | | | | | | | | | | | | | | | | | | | | | | | | | |  | | | | |  | | |
|  | | |  | | | | | | | | |  | | | | | | | | | | | |  | | | |  | | | | | | | | |  | | | | |  | | |
| Dose (μg) | | | BaCl | | | | | | | | | | | | | | | | | | | | | | | | | | | | | | | | | | | | | | | | | |
| 10 | | | 0 | | | | | | | | | 0 | | | | | | | | | | | | 0 | | | | 0 | | | | | | | | | 0 | | | | | 0 | | |
| 20 | | | 8,18 | | | | | | | | | 6,66 | | | | | | | | | | | | 5 | | | | 10,18 | | | | | | | | | 6,66 | | | | | 3 | | |
| 30 | | | 17,27 | | | | | | | | | 9,99 | | | | | | | | | | | | 10 | | | | 17,27 | | | | | | | | | 9,99 | | | | | 10 | | |
| 40 | | | 27,27 | | | | | | | | | 12,12 | | | | | | | | | | | | 20 | | | | 28,27 | | | | | | | | | 12,12 | | | | | 19 | | |
| 50 | | | 23,33 | | | | | | | | | 13,33 | | | | | | | | | | | | 25 | | | | 23,33 | | | | | | | | | 14 | | | | | 28,33 | | |
| 100 | | | 37,45 | | | | | | | | | 20,54 | | | | | | | | | | | | 35 | | | | 35,45 | | | | | | | | | 18,54 | | | | | 35 | | |
| 200 | | | 45,45 | | | | | | | | | 34,22 | | | | | | | | | | | | 40 | | | | 46,45 | | | | | | | | | 33,22 | | | | | 40 | | |
| 300 | | | 48,33 | | | | | | | | | 39,88 | | | | | | | | | | | | 45 | | | | 49,33 | | | | | | | | | 38,88 | | | | | 45 | | |
| 400 | | | 50 | | | | | | | | | 45,77 | | | | | | | | | | | | 50 | | | | 50 | | | | | | | | | 45,77 | | | | | 50 | | |
| 500 | | | 53,33 | | | | | | | | | 48,88 | | | | | | | | | | | | 50 | | | | 53,33 | | | | | | | | | 48,88 | | | | | 50 | | |
|  | | |  | | | | | | | | |  | | | | | | | | | | | |  | | | |  | | | | | | | | |  | | | | |  | | |
| the influence of EFCS in the absence or in the presence of Glibenclamide | | | | | | | | | | | | | | | | | | | | | | | | | | | | | | | | | | | | | | | | | |  | | |
|  |  | | | | | | | | |  | | | | | | | | | |  | | | | | | | | | |  | | | | | | | |  | | | |  | | |
| Dose (μg) | Glibenclamide | | | | | | | | | | | | | | | | | | | | | | | | | | | | | | | | | | | | | | | | | | | |
| 10 | 0 | | | | | | | | | 0 | | | | | | | | | | 5,66 | | | | | | | | | | 0 | | | | | | | | 7,66 | | | | 0 | | |
| 20 | 0 | | | | | | | | | 14,28 | | | | | | | | | | 7,33 | | | | | | | | | | 0 | | | | | | | | 15,28 | | | | 8,33 | | |
| 30 | 8,33 | | | | | | | | | 21,42 | | | | | | | | | | 12,5 | | | | | | | | | | 6,33 | | | | | | | | 23,42 | | | | 12,5 | | |
| 40 | 16,66 | | | | | | | | | 28,57 | | | | | | | | | | 15 | | | | | | | | | | 16,66 | | | | | | | | 30,57 | | | | 13 | | |
| 50 | 25 | | | | | | | | | 35,71 | | | | | | | | | | 17,5 | | | | | | | | | | 25 | | | | | | | | 37,71 | | | | 15,5 | | |
| 100 | 25 | | | | | | | | | 35,71 | | | | | | | | | | 20 | | | | | | | | | | 25 | | | | | | | | 37,71 | | | | 18 | | |
| 200 | 26,66 | | | | | | | | | 38,48 | | | | | | | | | | 20 | | | | | | | | | | 26,66 | | | | | | | | 40,48 | | | | 18 | | |
| 300 | 30,16 | | | | | | | | | 48,33 | | | | | | | | | | 30 | | | | | | | | | | 30,16 | | | | | | | | 50,33 | | | | 28 | | |
| 400 | 38,48 | | | | | | | | | 53,33 | | | | | | | | | | 35 | | | | | | | | | | 38,48 | | | | | | | | 55,33 | | | | 33 | | |
| 500 | 48,33 | | | | | | | | | 53,33 | | | | | | | | | | 45 | | | | | | | | | | 48,33 | | | | | | | | 54,33 | | | | 44 | | |
|  |  | | | | | | | | |  | | | | | | | | | |  | | | | | | | | | |  | | | | | | | |  | | | |  | | |
| the influence of EFCS in the absence or in the presence of L-NAME + Indo + TEA + Gly + BaCl | | | | | | | | | | | | | | | | | | | | | | | | | | | | | | | | | | | | | | | | | | | | |
|  | | | | | | | | |  | | | | | | | |  | | | | | | | | | |  | | | | | | | | |  | | | |  | | |  | |
| Dose (μg) | | | | | | | | | L-NAME + INDo + TEA + Gly + BaCl | | | | | | | | | | | | | | | | | | | | | | | | | | | | | | | | | | | |
| 10 | | | | | | | | | 1 | | | | | | | | 0,1 | | | | | | | | | | 0,1 | | | | | | | | | 0,1 | | | | 0,1 | | | 1 | |
| 20 | | | | | | | | | 1 | | | | | | | | 1 | | | | | | | | | | 1 | | | | | | | | | 1 | | | | 1 | | | 1 | |
| 30 | | | | | | | | | 1 | | | | | | | | 1 | | | | | | | | | | 1 | | | | | | | | | 1 | | | | 1 | | | 1 | |
| 40 | | | | | | | | | 1 | | | | | | | | 1 | | | | | | | | | | 1 | | | | | | | | | 1 | | | | 1 | | | 1 | |
| 50 | | | | | | | | | 1 | | | | | | | | 1 | | | | | | | | | | 1 | | | | | | | | | 1 | | | | 1 | | | 1 | |
| 100 | | | | | | | | | 1 | | | | | | | | 2,5 | | | | | | | | | | 1 | | | | | | | | | 1 | | | | 2,5 | | | 1 | |
| 200 | | | | | | | | | 2,5 | | | | | | | | 2,5 | | | | | | | | | | 2 | | | | | | | | | 2,5 | | | | 3 | | | 2,5 | |
| 300 | | | | | | | | | 2,5 | | | | | | | | 6 | | | | | | | | | | 5 | | | | | | | | | 2,5 | | | | 3,5 | | | 5,5 | |
| 400 | | | | | | | | | 5 | | | | | | | | 7 | | | | | | | | | | 7 | | | | | | | | | 6 | | | | 7 | | | 6 | |
| 500 | | | | | | | | | 7,5 | | | | | | | | 8 | | | | | | | | | | 8,5 | | | | | | | | | 6,5 | | | | 8 | | | 7,5 | |
|  | | | | | | | | |  | | | | | | | |  | | | | | | | | | |  | | | | | | | | |  | | | |  | | |  | |
